# Acetylcholine induces GABA release onto rod bipolar cells through heteromeric nicotinic receptors expressed in A17 amacrine cells

**DOI:** 10.3389/fncel.2015.00006

**Published:** 2015-02-09

**Authors:** Claudio Elgueta, Alex H. Vielma, Adrian G. Palacios, Oliver Schmachtenberg

**Affiliations:** ^1^Centro Interdisciplinario de Neurociencia de Valparaíso, Facultad de Ciencias, Universidad de ValparaísoValparaíso, Chile; ^2^Systemic and Cellular Neurophysiology, Institute of Physiology I, Albert-Ludwigs-UniversitätFreiburg, Germany

**Keywords:** acetylcholine, A17 amacrine cell, GABA, GABA receptors, nicotinic receptor, retina, rod bipolar cell, rod pathway

## Abstract

Acetylcholine (ACh) is a major retinal neurotransmitter that modulates visual processing through a large repertoire of cholinergic receptors expressed on different retinal cell types. ACh is released from starburst amacrine cells (SACs) under scotopic conditions, but its effects on cells of the rod pathway have not been investigated. Using whole-cell patch clamp recordings in slices of rat retina, we found that ACh application triggers GABA release onto rod bipolar (RB) cells. GABA was released from A17 amacrine cells and activated postsynaptic GABA_A_ and GABA_C_ receptors in RB cells. The sensitivity of ACh-induced currents to nicotinic ACh receptor (nAChR) antagonists (TMPH ~ mecamylamine > erysodine > DhβE > MLA) together with the differential potency of specific agonists to mimic ACh responses (cytisine >> RJR2403 ~ choline), suggest that A17 cells express heteromeric nAChRs containing the β_4_ subunit. Activation of nAChRs induced GABA release after Ca^2+^ accumulation in A17 cell dendrites and varicosities mediated by L-type voltage-gated calcium channels (VGCCs) and intracellular Ca^2+^ stores. Inhibition of acetylcholinesterase depolarized A17 cells and increased spontaneous inhibitory postsynaptic currents in RB cells, indicating that endogenous ACh enhances GABAergic inhibition of RB cells. Moreover, injection of neostigmine or cytisine reduced the b-wave of the scotopic flash electroretinogram (ERG), suggesting that cholinergic modulation of GABA release controls RB cell activity *in vivo*. These results describe a novel regulatory mechanism of RB cell inhibition and complement our understanding of the neuromodulatory control of retinal signal processing.

## Introduction

Nicotinic acetylcholine receptors are widely distributed throughout the central nervous system and play essential roles in learning, cognition and addiction (Dani and Bertrand, 2007). Central nAChRs are pentameric cationic channels assembled as homomers of α_7_–α_9_ subunits or by combinations of α_2_–α_6_ and β_2_–β_4_ subunits (Millar and Gotti, 2009). This heterogeneity endows nAChRs with different physiological and pharmacological properties and therefore diverse functional roles in neuronal networks (Mansvelder et al., 2006; Dani and Bertrand, 2007; Albuquerque et al., 2009).

In the mammalian retina, ACh is synthesized and released from starburst amacrine cells (SACs) that form narrowly defined cholinergic plexuses in the inner plexiform layer (IPL) (Voigt, 1986). Release of ACh may activate different classes of cholinergic receptors present in bipolar, amacrine and ganglion cells (AC and GCs) (Keyser et al., 2000; Dmitrieva et al., 2001, 2003, 2007; Moretti et al., 2004; Marritt et al., 2005; Strang et al., 2010). Indeed, functional nAChRs are expressed in GCs that stratify close to SAC dendrites (Kittila and Massey, 1997; Fried et al., 2005; Reed et al., 2005; Strang et al., 2007; Briggman et al., 2011) as well as in cells whose processes are located far from these cholinergic bands (Masland and Ames, 1976; Ariel and Daw, 1982; Schmidt et al., 1987; Strang et al., 2003, 2005). Activation of nAChRs also modulates the ON bipolar cell-dependent b-wave of the electroretinogram (ERG) (Jurklies et al., 1996; Varghese et al., 2011; Moyano et al., 2013), suggesting that ACh may influence signal transmission at stages preceding GC activation, but its specific targets and mechanisms of action remain largely unknown.

ACh release occurs under a broad range of illuminations (Masland and Livingstone, 1976; Massey and Neal, 1978, 1979b; O'Malley and Masland, 1993), including scotopic conditions when luminous signals detected by rods are mainly processed by the classic rod pathway. The first steps in this circuit involve the sequential activation of two dedicated cell types, RB and AII-ACs. The latter form chemical and electric synapses with cone bipolar cells to convey rod signals to GCs (Bloomfield and Dacheux, 2001; Wässle, 2004). Glutamate release from RB to AII cells remains tightly controlled by numerous reciprocal GABAergic synapses between A17 AC and RB cell axons (Hartveit, 1999; Singer and Diamond, 2003; Chávez et al., 2006), providing stability and enhanced temporal resolution at the RB-AII cell synapse (Nakatsuka and Hamasaki, 1985; Dong and Hare, 2002a,b).

In the present study we analyzed the influence of ACh on signal processing in the classic rod pathway. We have found that GABA release from A17 cells can be elicited by activation of nAChRs and that this modulation regulates RB cell activity, a new mechanism by which retinal circuits shape visual responses under low light conditions.

## Materials and methods

All experiments were performed on 30–50 day-old Sprague Dawley rats irrespective of sex. The rats, born and raised in the animal facility of the University of Valparaiso (Animal Welfare Assurance NIH A5823-01), were held at 20–25°C under a 12 h photoperiod with water and food *ad libitum*. The experimental procedures were approved by the bioethics committee of the University of Valparaiso and in accordance with the bioethics regulation of the Chilean Research Council (CONICYT).

### Electrophysiology

After a period of dark adaptation (≥1 h), rats were decapitated under deep anesthesia and their eyes were removed and submerged in ACSF containing (mM) 119 NaCl, 23 NaHCO_3_, 1.25 NaH_2_PO_4_, 2.5 KCl, 2.5 CaCl_2_, 1.5 MgCl_2_, 20 Glucose, 2 Na-Pyruvate, 1 ascorbic acid (bubbled with a mixture of 95% O_2_–5% CO_2_). The retina was isolated and embedded in low melting point agarose (3%), glued to a vibratome stage and sections of ~200 μm thickness were obtained. All slicing procedures were performed under dim red light and sections were kept in the dark at room temperature. Retinal slices were then transferred to a recording chamber in which they were superfused with ACSF 1–2 ml/min (~30°C) supplemented with strychnine (2 μM) to block glycine receptors. Cells were selected using a BX51WI microscope (Olympus, Japan) placed in a light tight enclosure and equipped with infrared differential interference contrast. Patch pipettes were pulled from borosilicate glass and had resistances of ~5 or ~10 MΩ (for AC and RB cells recordings, respectively) when filled with an intracellular solution containing (mM) 125 cesium-methanesulfonate, 10 HEPES, 5 EGTA, 6 Na_2_ATP, 0.4 GTP, 15 TEA-Cl, 1 MgSO_4_ (pH 7.4; osmolarity ~294 mOsm) and 1% Lucifer Yellow (Sigma Aldrich, St. Louis, MO, USA) or Alexa Fluor 488 (Invitrogen, USA). Signals were recorded using a PC-501A (Warner Instruments, Hamden, USA) or an EPC7-plus (HEKA Elektronik, Lambrecht, Germany) amplifier, digitized at 20 kHz (PCI-6221, National Instruments, Austin, TX, USA) and stored using custom software written in IGOR PRO (Wavemetrics, Lake Oswego, OR, USA). Series resistance was below 20 or 30 MΩ for AC and RB cells respectively, and left uncompensated. Liquid junction potential was calculated to be 10 mV and corrected off-line. ACs were kept at a holding membrane potential (V_hold_) of −60 mV while IPSCs in RB cells were recorded at 0 mV. Cells were morphologically identified at the end of the experiment using the fluorescence image obtained with a digital camera (DS-MBWc, Nikon, Japan).

### Calcium imaging

To record calcium signals from A17 cells, glass pipettes were front-filled with normal intracellular solution and back-filled with the same solution supplemented with the calcium indicator Oregon Green 488 BAPTA-1 (OGB-1, 100–150 μM, Molecular Probes, Eugene, OR, USA). After ~15 min, images were obtained using a CooLed illumination system (Cooledinc, Traverse City, MI, USA) and a Sensicam QE digital camera (Cooke Corp., Romulus, MI, USA). Data was acquired at 125 Hz and analyzed using custom software written in IGOR PRO.

### Electroretinography

Methods used for electroretinography have been described previously (Vielma et al., 2010). Briefly, animals were dark adapted for 2 h and anesthetized by halothane inhalation followed by injection of ketamine (40 mg/kg) and xylazine (4 mg/kg). Pupils were dilated using atropine (1%) and lidocaine (1%) was used as a topic anesthetic. Signals were recorded using a silver chloride ring electrode on the cornea and a subcutaneous platinum electrode in the eyelids as a reference. Animals were stimulated for 10 ms with a light spot of 1.5 mm of diameter, illuminance of 1.9 log scot cd s m^−2^ and wavelength of 500 nm. After recording control responses, animals were injected with the tested drugs dissolved in PBS using a 27-gauge needle through the ora serrata into the vitreous. Drug concentrations in the eye were calculated considering an average vitreal volume of 0.15 ml. ERG responses were measured again after ~10 min. Rats were finally sacrificed by an overdose of halothane.

### Drugs and solutions

ACh, choline, neostigmine, verapamil, nifedipine, 4-Chloro-methyl-cresol, and ruthenium red were purchased from Sigma (St. Louis, MO, USA). Mecamylamine, dihydro-β-erythroidine, phenserine, TPMPA, SR95531, methyllycaconitine, tetramethylpiperidine-4-yl-heptanoate, tetrodotoxin (TTX), cyclopiazonic acid and strychnine were obtained from TOCRIS (Bristol, UK). Erysodine and cytisine were synthesized and kindly provided by Dr. Patricio Iturriaga from the Chemistry department of the Universidad de Chile. All drugs were dissolved as stock solutions in bi-distilled water or DMSO, and were diluted to the final concentration in control ACSF before the experiment. For localized puff applications, agonists were dissolved in HEPES-buffered ACSF in which NaHCO_3_ was substituted for HEPES. Puffs were applied into the (IPL) from glass pipettes with ~1.5 μm tip of inner diameter, using a custom-built picospritzer with 5 psi standard pressure. Specific nAChR agonists were loaded into one compartment of a puffer pipette pulled from borosilicate theta glass (World precision instruments, Sarasota, FL, USA) while the other was loaded with ACh, achieving similar pressure ejection conditions for both compounds. Puff applications were separated by 3–6 min. Drugs added to the bath were superfused for a minimum time of 10 min before evaluating its effects. In a subset of experiments, tested drugs were washed-out (>30 min) and recovery of responses from the treatment was evaluated (See Supplementary Table 1).

### Data analysis

**Figure 1:**
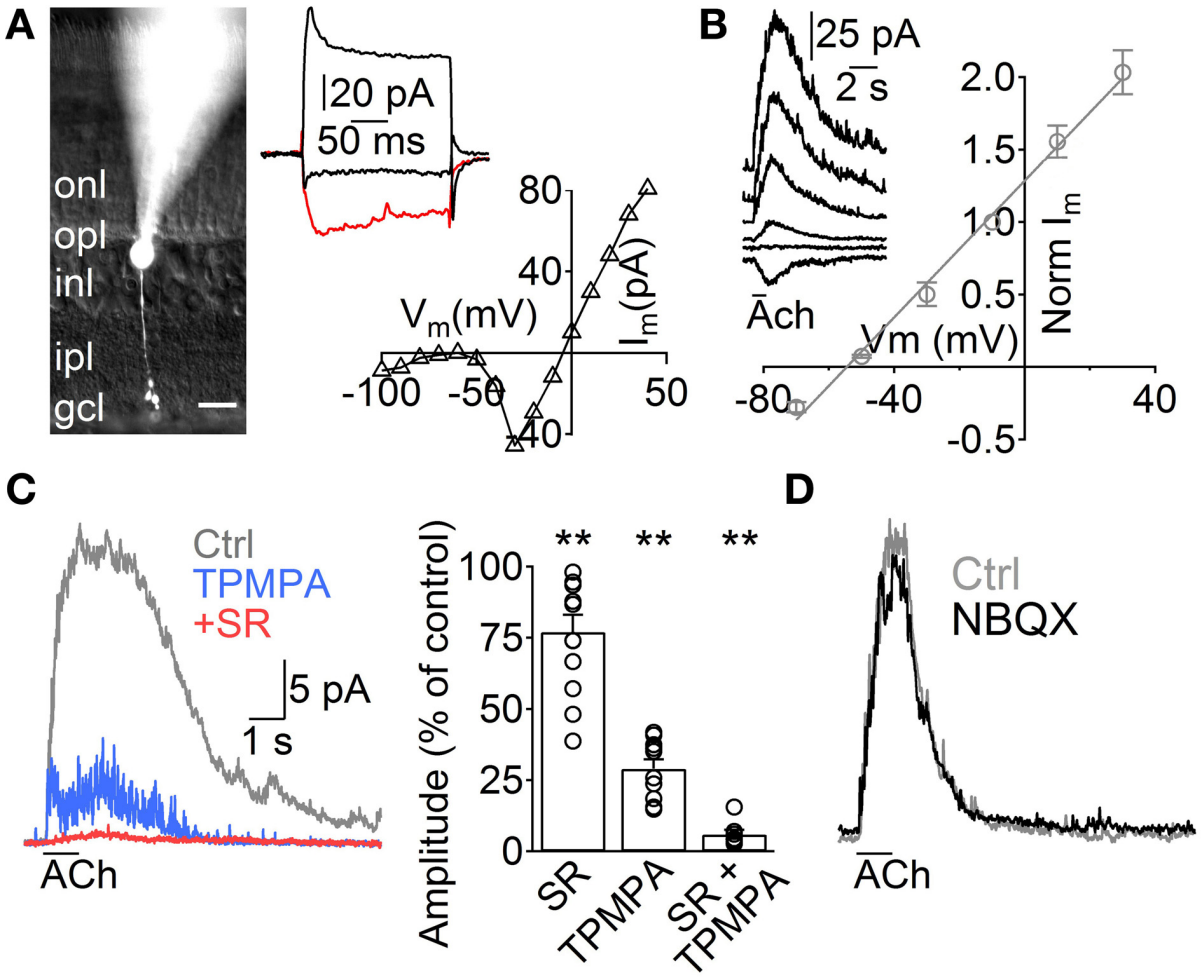
**ACh induces GABA release from A17 cells onto rod bipolar cells**. **(A)** Left, image of a rod bipolar (RB) cell filled with Lucifer yellow during whole-cell patch clamp recordings. Scale bar indicates 10 μm. Right top, current responses from the cell in the left to voltage steps from −60 to −100, −20 (red trace) and 40 mV. Right bottom, current-voltage relationship for the same cell (10 mV voltage steps from −120 to 40 mV). **(B)** Left, representative current responses of RB cells to puff applications of ACh (1 mM, 1 s) at different holding potentials. Right, normalized amplitude of ACh responses in RB cells plotted against the holding potential (*n* = 4). **(C)** Traces showing the effect of GABA_C_ receptor–specific antagonist TPMPA (50 μM) and GABA_A_ antagonist SR95531 (SR, 10 μM) on ACh-evoked IPSCs in RB cells. (V_hold_ = 0 mV). Right, bar plot summarizes the effect of both GABA receptor antagonists applied separately or combined on ACh-induced responses. **(D)** Traces depict the lack of effect of AMPA/Kainate receptor antagonist application (NBQX 5 μM) in ACh-induced IPSCs (see text). Two-tailed paired *t*-test, ^**^p < 0.01.

Data analysis was performed in IGOR PRO. Input resistance (R_in_) was obtained by measuring the inverse slope of a linear fit to the current-voltage plots (10 mV steps from −120 to −80 mV). Peak amplitude was measured after low-pass filtering at 1 kHz for electrophysiological data or after filtering at 25 Hz and subtraction of background fluorescence for Ca^2+^ imaging signals. Electrical charge moved during a response was calculated as the integral of the baseline-subtracted response. Reversal potential of ACh evoked currents was calculated as the intercept of a line-fit of the peak amplitude values plotted against the corresponding V_hold_. Decay of evoked responses was fitted to a mono-exponential function. Frequency of spontaneous postsynaptic currents was quantified from 2–4 min recordings obtained during control conditions and immediately after arrival of the tested agents using automatic detection algorithms and manually reviewing the obtained results (Mini Analysis, Synaptosoft Inc., Decatur, GA, USA). Statistical analysis was performed using SigmaPlot (Systat software). After evaluation for normality using Shapiro-Wilk normality test, statistical significance was evaluated using two-tailed paired student's *t*-test. In **Figures 6E,F**, significant differences were tested using a repeated measures ANOVA and Bonferroni-corrected paired comparisons. Except indicated, all experiments were compared to their own control. For comparing multiple treatments, a One-Way ANOVA or a Kruskal-Wallis One-Way ANOVA on ranks on the normalized changes was used, followed by *post hoc* pairwise comparison using Dunn's method. In Figures 1C, 3G, 4D, 5C, 6C bar plots represent percentage of control ± standard error. In **Figures 6H,I** and Figure S1G, bar plots depict mean frequency ± standard error. Circles in bar plots show individual experiments. ^*^indicates *p* < 0.05, ^**^p < 0.01, and ^***^p < 0.001. Traces were filtered at 100 Hz for display purposes.

## Results

### Acetylcholine induces GABAergic signaling onto RB cells

To study the influence of ACh on the rod pathway, we started by performing voltage clamp experiments in RB cells. These cells have their cell body generally located in the outer part of the INL and show sustained inward currents upon depolarization largely mediated by L-type VGCCs (Protti and Llano, 1998), allowing confirmation of their identity under whole cell voltage clamp (Figure 1A, right). *Post hoc* morphological examination revealed the characteristic RB cell features (Chávez et al., 2006), an axon traversing the entire IPL that narrowly extends axonal boutons close to the GC layer (Figure 1A, left). Application of ACh (1 mM, 1 s) to the middle of the IPL induced outward currents in all RB cells tested (V_hold_ 0 mV, *n* = 129), which had a reversal potential close to the Cl^−^ equilibrium potential (E_Cl_ = −52.1 mV, E_rev_ = −54.5 mV, *n* = 5, Figure 1B). Pharmacological analysis revealed that ACh-evoked currents were generated by the activation of GABA_A_ (77 ± 6% of control amplitude after SR95531 10 μM, *n* = 12, *p* = 0.008) and GABA_C_ receptors (29 ± 3.3% of control after TPMPA 50 μM, *n* = 10, *p* = 0.0025; 6.2 ±1.4% of control with SR95531 and TPMPA combined, *n* = 8, *p* = 0.005, Figure 1C). Blocking AMPA and kainate receptors did not have a significant effect on the responses to ACh (NBQX 5 μM, 94.2 ± 2.9% of control, *n* = 5, *p* = 0.2, Figure 1D), suggesting they were generated by direct cholinergic activation of ACs presynaptic to RB cells.

### A17 cells mediate ACh-induced GABA release onto RB cells

**Figure 2:**
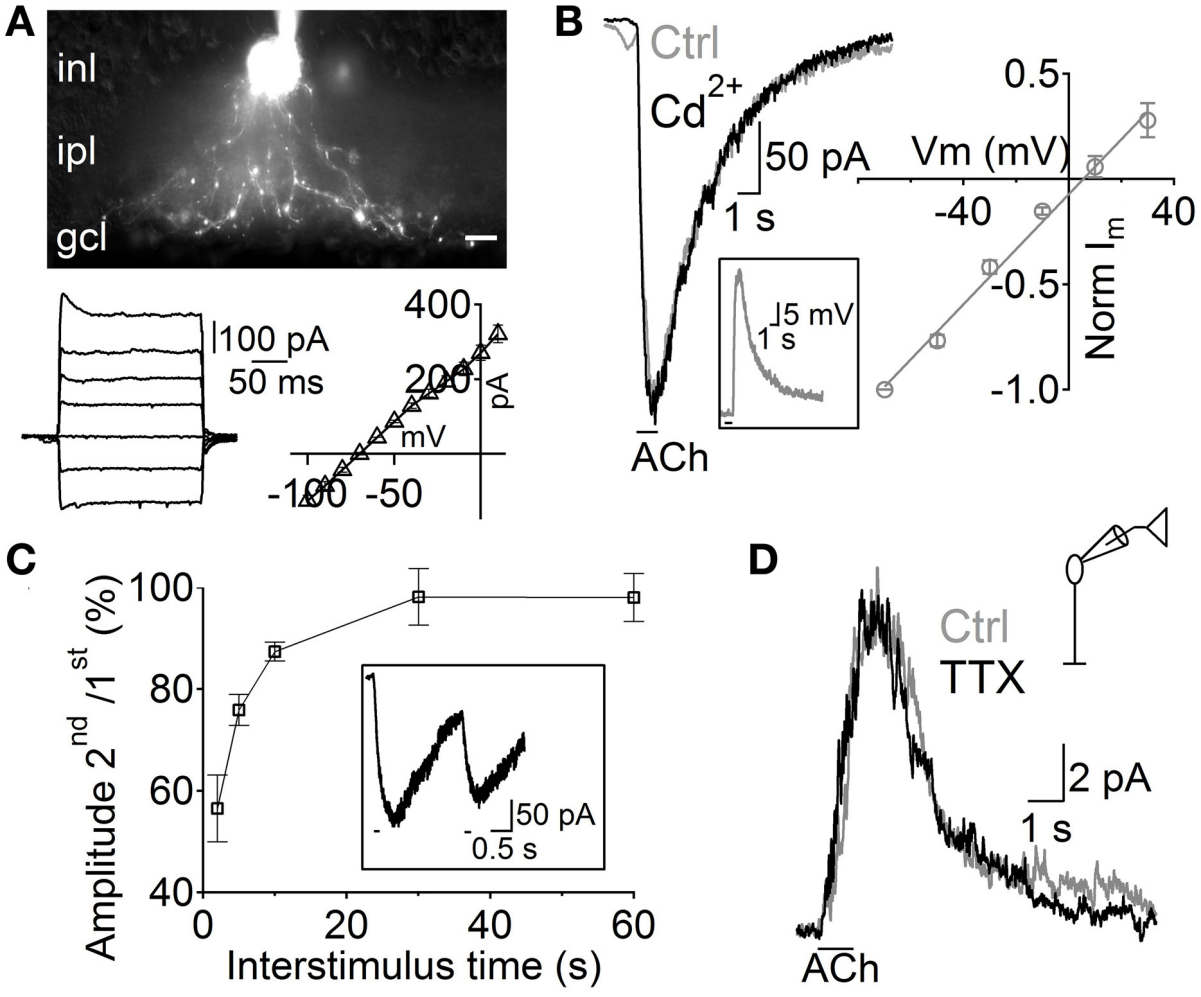
**Depolarization of A17 cells mediates acetylcholine evoked IPSCs in RB cells. (A)** top, image of an A17 cell filled with Lucifer yellow during whole-cell patch clamp. Scale bar indicates 10 μm. Bottom, (left) responses of the A17 cell to voltage steps from −100 to 20 mV (20 mV steps) and (right) plot of its current-voltage relationship. **(B)** Representative trace of ACh-evoked currents in A17 cells (V_hold_ – 60 mV) which were unaffected by calcium channel blockage with Cd^2+^ (200 μM). Inset shows the response of an A17 cell to ACh in current clamp mode. Right, normalized amplitude of ACh responses in A17 cells plotted against holding potential (*n* = 4). **(C)** Summary of paired responses to ACh (1 mM, 100 ms) with different inter-stimulus intervals. Inset shows the response to a paired stimulus separated by 2 s. **(D)** ACh-evoked IPSCs in rod bipolar cells were insensitive to perfusion of tetrodotoxin (TTX 1 μM, *n* = 7) suggesting that GABA is released from A17 cells (Chávez et al., 2010).

Although RB cells receive inputs from different GABAergic ACs, almost half of their inhibitory axonal contacts are reciprocal synapses with A17 cells (Strettoi et al., 1990; Kim et al., 1998). Therefore, we tested the possibility that these ACs generated the GABAergic IPSCs evoked by ACh in RB cells. A17 cells were selected in retinal slices by aiming at large oval-shaped cell bodies located in the inner part of the inner nuclear layer. During voltage clamp recordings, the low input resistance (224 ± 11 MΩ, *n* = 99) and nearly linear current-voltage relationship (Figure 2A, bottom) of A17 cells provided a reliable indicator of cell identity. Fluorescent images confirmed our physiological identification and showed the main morphological properties of A17 cells (Menger and Wässle, 2000), namely the presence of multiple thin dendrites bearing varicosities that radially extend toward the GCL border (Figure 2A). In all A17 cells tested, pulsed applications of ACh to the IPL induced strong inward currents (average amplitude −215 ± 12 pA, V_hold_ −60 mV, *n* = 98, Figure 2B) and a mean depolarization of 33.6 ± 3.7 mV in current clamp recordings (*n* = 5, membrane resting potential −61.6 ± 1 mV, Figure 2B, inset). Currents evoked by ACh reversed at 5.5 mV (*n* = 4; Figure 2B, right) and were insensitive to synaptic block by divalent VGCC blockers (100.9 ± 5.7% of control response with Cd^2+^ 200 μM, *n* = 5, *p* = 1, Figure 2B left; 98.7 ± 4.1% of control with Co^2+^ 1 mM, *n* = 4, *p* = 0.65, Figure S1A) or inhibition of AMPA/kainate receptors (NBQX 5 μM, 116.5 ± 8.6%, *n* = 3, *p* = 0.42, Figure S1B), demonstrating their postsynaptic origin. ACh-evoked responses only mildly desensitize at the concentrations tested, as shown by the fact that a second application of ACh after a 2 s interval triggered currents with an amplitude of 56.5 ± 6.6% of the control response (*n* = 3; Figure 2C).

Interestingly, other populations of ACs also displayed inward currents when either ACh or nicotine was applied (69 out of 206 non-A17 ACs tested, data not shown). These cells formed a heterogeneous population displaying different morphological and physiological properties indicating that cholinergic modulation of inhibition is a widespread phenomenon in the rat retina. However, isolation of GABAergic IPSCs from A17 onto RB cells with TTX (1 μM, Chávez et al., 2006) left postsynaptic ACh responses unchanged (93 ± 12% of control, *p* = 0.2, *n* = 6; Figure 2D). In summary, we have found that ACh-evoked GABAergic IPSCs in RB cells are mostly generated by GABA released from A17 cells after the activation of functional cholinergic receptors.

### Pharmacology of A17 cell nAChRs

**Figure 3:**
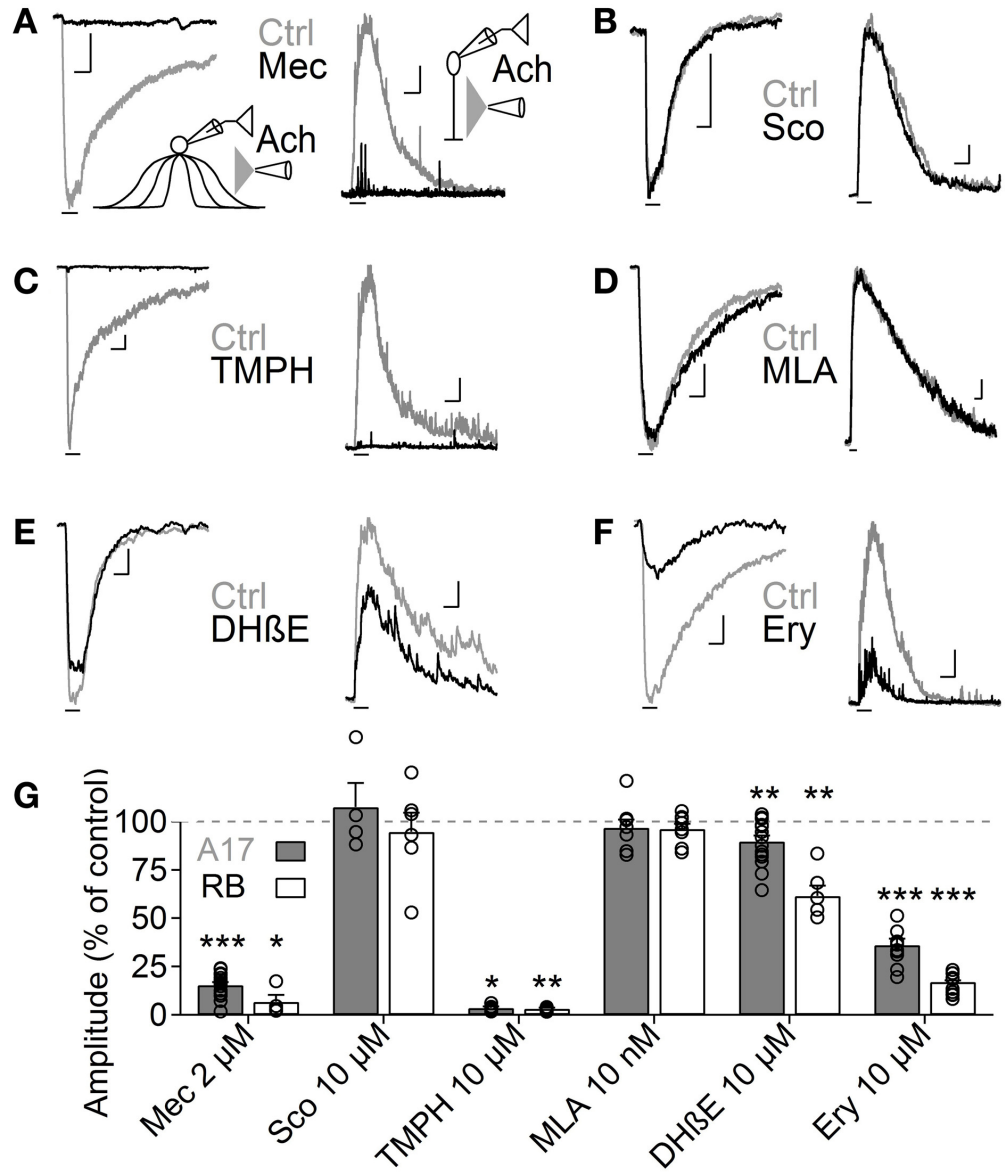
**Heteromeric nAChRs cells mediate ACh responses in A17 and RB cells**. **(A–F)** Representative traces of current responses induced by localized puffs of ACh (1 mM, 1 s) at the IPL in A17 (left) and in RB cells (right) in control conditions and in the presence of **(A)** the nicotinic receptor blocker mecamylamine (Mec 2 μM), **(B)** muscarinic receptor antagonist scopolamine (Sco 10 μM), **(C)** heteromeric nAChR antagonist tetramethylpiperidine-4-yl-heptanoate (TMPH 10 μM), **(D)** homomeric nAChR antagonist methylcaconitine (MLA 10 nM) or α_4_β_2_ antagonists **(E)** dihydro-β-erythroiodine (DHβE 10 μM) and **(F)** erysodine (Ery 10 μM). Vertical scale bars represent 50 pA for A17 cell responses and 5 pA for RB cell traces, and horizontal bars depict 1 s of time. **(G)** Bar graph summarizing the effects of cholinergic receptor antagonists on the ACh-induced currents in A17 (gray bars) and RB cells (white bars). Two-tailed paired *t*-tests, ^*^p < 0.05, ^**^p < 0.01, and ^***^p < 0.001. For A17 cells, significant differences were observed between Mec and TMPH if tested vs. the effects of Sco, MLA or DHβE. For RB cells, differences were significant when comparing the influence of Mec and TMPH vs. Sco or MLA (One-Way ANOVA on ranks and Dunn's method for multiple comparisons).

ACh is the natural agonist of metabotropic and ionotropic cholinergic receptors. Given that both can be found in the mammalian retina (Wassélius et al., 1998; Keyser et al., 2000; Marritt et al., 2005), we asked which of these receptor types mediates the release of GABA from A17 cells. In agreement with their cationic nature, ACh-induced currents in A17 cells were effectively reduced by the general nAChR antagonist mecamylamine (Mec 2 μM, 15.4 ± 1.5% of control, *n* = 17, *p* = 0.00004) but unaffected by the muscarinic antagonist scopolamine (Sco 10 μM, 107.4 ± 12.4% of control, *n* = 4, *p* = 0.58; Figures 3A,B left, G). Moreover, nicotine also effectively elicited inward currents in A17 cells (Nicotine 1 mM, 500 ms, −199.6 ± 33 pA, *n* = 10, **Figure 5D**, middle). Consequently, IPSCs evoked after ACh application in RB cells were completely abolished by mecamylamine (6.7 ± 3.6% of control, *n* = 4, *p* = 0.04) while scopolamine had no significant effect (94.8 ± 9.9% of control, *n* = 6, *p* = 0.9; Figures 3A,B right, G). Thus, A17 cells of the rat retina express functional nAChRs.

**Figure 4:**
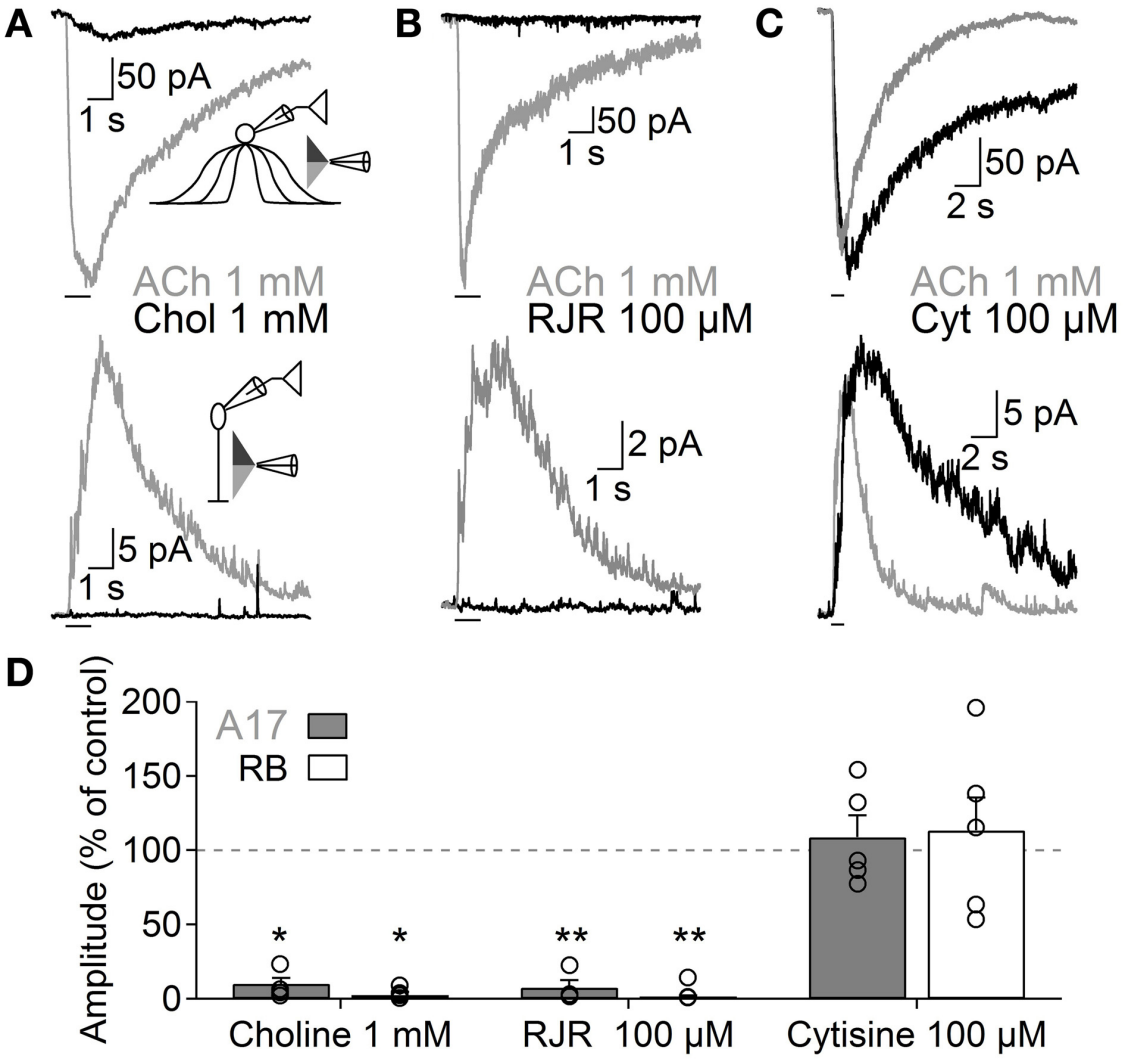
**Functional β_4_-containing nAChRs are expressed in A17 cells and mediate ACh-evoked GABA release onto RB cells**. Representative traces of whole-voltage clamp recordings during localized application of ACh (1 mM, 1 s) or nicotinic agonists specific for **(A)** homomeric (choline 1 mM, 1 s, top), **(B)** α_4_β_2_ (RJR-2403 100 μM, 1 s, middle) or **(C)** β_4_ subunit-containing nAChRs (cytisine 100 μM, 1 s, bottom) in A17 (top) or RB cells (bottom). All experiments were performed using double-barreled puffer pipettes. **(D)** Bar plot summarizing the normalized effects of specific nicotinic agonists on A17 (gray solid bars) and RB (with bars) cells. Two-tailed paired *t*-test, ^*^p < 0.05 and ^**^p < 0.01. The normalized average effect of cytisine was significantly different when compared to choline or RJR-2403 in A17 (One-Way ANOVA with Bonferroni-adjusted pairwise comparisons) and RB cells (One-Way ANOVA on ranks with *post hoc* Dunn's test).

Nicotinic receptors are assembled by different combinations of α and β subunits. The precise subunit composition defines important receptor properties such as ligand affinity, channel kinetics and ionic selectivity (Alkondon and Albuquerque, 1993), greatly affecting the influence that nAChR activation will have in cellular function. To investigate which nAChR subtypes are expressed in A17 cells and mediate postsynaptic responses in RB cells, we tested the effects of specific nicotinic pharmacological agents. Perfusion of an antagonist specific for neuronal heteromeric nAChRs (tetramethylpiperidine-4-yl-heptanoate [TMPH] 10 μM, Damaj et al., 2005; Papke et al., 2005) abolished ACh responses in both cell types (3.6 ± 0.7% of control in A17 cells, *n* = 5, *p* = 0.02; 3.2 ± 0.4% of control in RB cells, *p* = 0.008, *n* = 5, Figures 3C,G), while homomeric nAChR-specific antagonist methyllycaconitine (MLA) did not produce a significant effect neither in A17 nor in RB cells (10 nM; 96.8 ± 4.2 and 96.7 ± 2.7% of control responses, *p* = 0.25 and 0.17 respectively, *n* = 8 for both cells types, Figures 3D,G). One of the most commonly expressed heteromeric nAChRs in the CNS is the α_4_β_2_ subtype (Dani and Bertrand, 2007). The prototypical α_4_β_2_ nAChR antagonist dihydro-β-erythroiodine (DHβE 10 μM, Harvey et al., 1996) produced a small albeit significant reduction of ACh-induced currents in A17 cells (89.7 ± 3% of control, *n* = 15, *p* = 0.009) and a marked decrease in RB cell responses (61 ± 5.3% of control, *n* = 6, *p* = 0.003; Figures 3E,G). Erysodine (10 μM), another alkaloid with high affinity for α_4_β_2_ nAChRs (Decker and Anderson, 1995; Iturriaga-Vásquez et al., 2010), had a more pronounced effect in A17 and RB cells (36.1 ± 3.3% of control in A17 cells, *n* = 10, *p* = 0.0002; 17 ± 0.8% of control in RB cells, *n* = 10, *p* = 0.00003; Figures 3F,G) but did not completely block the response to ACh. Pharmacology of nicotinic receptors in A17 cells was further investigated by comparing ACh-evoked currents with those induced by specific nicotinic agonists. Choline (Chol, 1 mM), a byproduct of ACh cleavage and an α_7_-nAChR-specific ligand (Alkondon and Pereira, 1999), produced only a marginal response in A17 cells and had no discernible effect in RB cells (10.1 ± 3.9 and 2.5 ± 2% of ACh-evoked responses respectively, *n* = 5 for both cell types, *p* = 0.03 and 0.017; Figures 4A,D). Likewise, an agonist with specificity for α_4_β_2_ nAChRs (RJR-2403 100 μM, Papke et al., 2000) failed to activate either A17 or RB cells (7.5 ± 5% and 1.7 ± 0.6% of ACh response, *n* = 4 and 3, *p* = 0.009 and 0.004, respectively; Figures 4B,D). On the contrary, the β_4_ subunit-specific nicotinic agonist cytisine (100 μM; Luetje and Patrick, 1991; Papke and Heinemann, 1994) induced currents in A17 and RB cells comparable to those evoked by ACh (108.7 ± 14.7% and 113.3 ± 22.1% of ACh responses respectively, *n* = 5 for both cell types, *p* = 0.58 and 0.97; Figures 4C,D), although with a slower decay (tau 3.9 ± 0.3 vs. 28.7 ± 4.9 s, for ACh vs. cytisine-evoked currents in A17 cells, *n* = 5, *p* = 0.047). In summary, ACh responses in both cell types have a comparable pharmacological profile, further supporting that A17 cells through activation of heteromeric nAChRs, provide the majority of the ACh-triggered GABA release onto RB cells.

### Mechanisms of ACh-evoked GABA release

**Figure 5:**
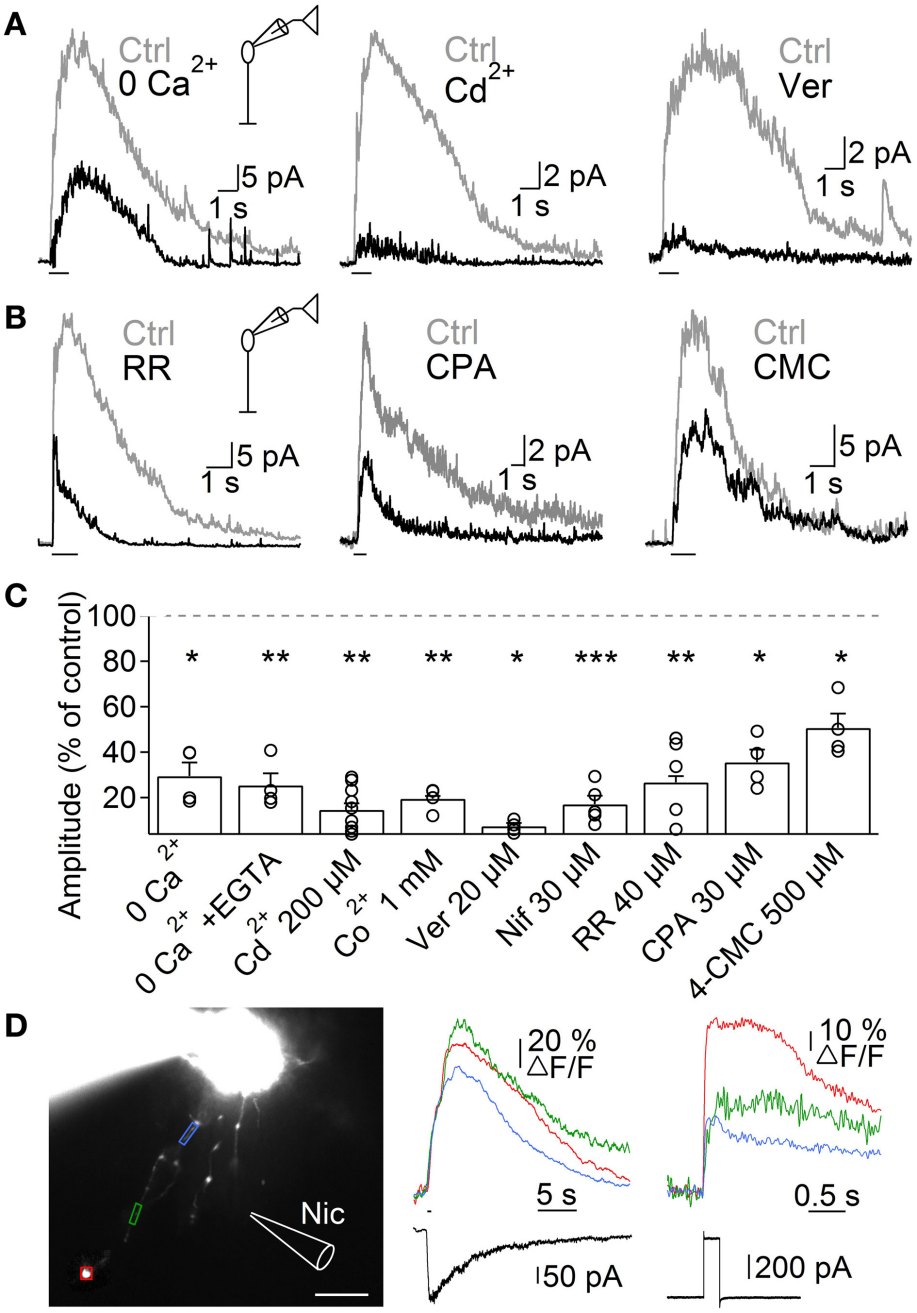
**Mechanisms of ACh-induced GABA release from A17 cells**. **(A)** Representative traces of ACh-evoked IPSCs in RB cells (1 mM, 1 s) in control conditions and after (left) removing extracellular Ca^2+^, (middle) applying the unspecific VGCC blocker Cd^2+^ (200 μM) or (right) after specific blockade of L-type VGCCs with verapamil (20 μM). **(B)** Traces showing the effects of disrupting intracellular calcium signaling by (left) a blocker of ryanodine receptor channels (ruthenium red, RR 40 μM), (middle) an inhibitor of the endoplasmic reticulum Ca^2+^-ATPase pump (cyclopiazonic acid, CPA 30 μM) and (right) an agonist of ryanodine receptors (4-chloro-m-cresol, 4-CMC 500 μM) on the responses to ACh in RB cells. **(C)** Bar graph displaying normalized average ACh response amplitudes in RB cells after perfusion with solutions affecting presynaptic calcium dynamics. ^*^p < 0.05, ^**^p < 0.01, and ^***^p < 0.001, for two-tailed paired *t*-tests. Differences between groups were significant when verapamil was compared with CPA or 4-CMC, and when Cd^2+^ is compared with 4-CMC (One-Way ANOVA on ranks with *post hoc* Dunn's test). **(D)** Left, montage of images from an A17 cell filled with 100 μM OGB-1 (scale bar 10 μm). Analyzed regions of interest are denoted by colored frames and correspond to Ca^2+^ responses traces after (middle) puff application of nicotine (Nic 1 mM, 500 ms) or (right) after a 60 mV voltage step of 200 ms duration. Bottom, black traces show current responses after the respective stimulation paradigms.

Next, we investigated the mechanisms through which nAChR activation induces Ca^2+^ entry into A17 cells, by studying ACh-evoked IPSCs in RB cells. Perfusion with Ca^2+^-free solutions importantly reduced the amplitude of ACh-evoked IPSCs in RB cells (0 Ca^2+^, 29.5 ± 6% of control, *n* = 4, *p* = 0.015, Figures 5A left, C; 0 Ca^2+^ + 1 mM EGTA, 25.3 ± 5.2% of control, *n* = 4, *p* = 0.003, Figures S1C, 5C). Because heteromeric nAChRs form channels with relatively low Ca^2+^ permeability (Fucile, 2004), Ca^2+^ influx should be provided by a different membrane conductance. Indeed, general blockers of VGCCs effectively reduced the response of RB cells to ACh (Cd^2+^ 200 μM, 14.5 ± 2.8% of control, *n* = 9, *p* = 0.001, Figures 5A middle, C; Co^2+^ 1 mM, 19.5 ± 1.2% of control, *n* = 4, *p* = 0.01, Figures S1D, 5C). More specifically, inhibition of L-type VGCCs, which are known to be present in synaptic varicosities of A17 cells (Grimes et al., 2009), almost completely abolished ACh responses (verapamil 20 μM, 7.3 ± 1.4% of control, *n* = 5, *p* = 0.02, Figures 5A right, C; nifedipine 30 μM, 17 ± 3.7% of control, *n* = 5, *p* = 0.0007, Figures S1E, 5C). This indicates a prevalent role of L-type VGCCs in cholinergic release of GABA from A17 cells, contrary to their limited participation when glutamate is the excitatory neurotransmitter (verapamil 20 μM, 91.8 ± 4.1% of control responses to glutamate [200 μM, 200 ms], *n* = 3, Figure S1F; see also Chávez et al., 2006; Grimes et al., 2009). On the other hand, Ca^2+^-induced Ca^2+^ release (CICR) from intracellular stores could also contribute by enhancing the cytoplasmatic Ca^2+^ concentration after an initial influx of the ion, as reported for glutamate-evoked GABA release from A17 cells (Chávez et al., 2006; Chávez and Diamond, 2008). Indeed, perfusion with an antagonist of ryanodine receptors produced a significant decrease in the amplitude of IPSCs triggered by ACh (ruthenium red, RR 40 μM, 26.7 ± 3% of control, *n* = 5, *p* = 0.002; Figures 5B left, C). Consistently, depletion of Ca^2+^ from the endoplasmic reticulum using the Ca^2+^-ATPase antagonist cyclopiazonic acid (CPA 30 μM) or the ryanodine receptors agonist 4-chloro-methyl-cresol (4-CMC 500 μM) significantly diminished IPSCs evoked by ACh in RB cells (35.5 ± 5.6% and 50.4 ± 6.3% of control response, *p* = 0.043 and 0.049, respectively, *n* = 4 for both conditions; Figures 5B,C).

To further confirm that activation of nAChRs induced Ca^2+^ accumulation in these ACs, we loaded A17 cells with the high affinity Ca^2+^-sensitive dye OGB-1, and imaged the response to nicotine puff applications. Nicotine generated strong and long lasting calcium signals (Figure 5D) that were significantly larger when measured at regions of interest that included dendritic varicosities compared to portions devoid of them (ΔF/F = 42 ± 9.4 vs. 23.7 ± 8.2%, *n* = 9, *p* = 0.004; Figure 5D, middle). Nicotine-induced calcium transients were similar in amplitude to those evoked by a depolarization step (60 mV, 200 ms, 44.8 ± 14.8 vs. 46.2 ± 8.9% of ΔF/F, *n* = 5, Figure 5D, right), indicating that activation of nAChRs has a strong depolarizing effect.

Altogether, these findings support the notion that nAChR-mediated depolarization of A17 cells activates L-type VGCCs and CICR, resulting in Ca^2+^ accumulation that finally drives GABA release onto RB cells.

### GABA release from A17 cells is modulated by endogenous ACh in vitro and in vivo

**Figure 6:**
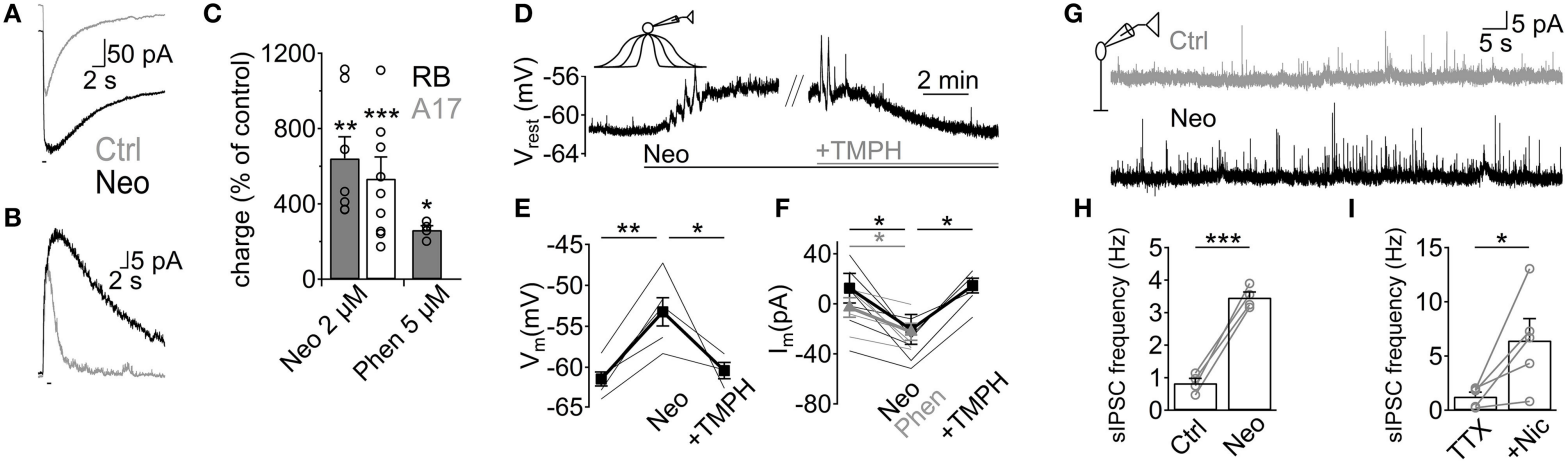
**Endogenous ACh enhances GABA release from A17 cells and increases inhibition in RB cells**. **(A,B)** Traces show the effect of the AChE inhibitor neostigmine (Neo, 2 μM) on responses to ACh (1 mM, 1 s) in **(A)** A17 cells and **(B)** RB cells. **(C)** Bar plot summing up the effects of neostigmine or phenserine (Phen, 5 μM) application on the electrical charge transferred after ACh stimulation. **(D)** In A17 cells, perfusion of neostigmine (2 μM) produced a long lasting depolarization that could be reverted by blocking nAChRs with TMPH (10 μM). Horizontal bars below traces show duration of both treatments. **(E)** Summary of the changes in resting membrane potential induced by an initial perfusion of Neo and subsequent addition of TMPH. **(F)** In a different subset of A17 cells, the holding current (V_hold_ = −60 mV) was measured in control conditions, after adding AChE inhibitors Neo or Phen, and after TMPH application. Thin lines denote individual cells and thick lines display average values. Gray lines correspond to experiments with Phen, black lines show experiments using Neo. **(G)** representative traces showing the increase in the frequency of spontaneous IPSCs after neostigmine perfusion in RB cells. **(H)** Bar plot summarizing the enhancement in IPSC frequency by Neo. **(I)** Summary plot showing the marked increase in frequency of IPSCs after perfusion of nicotine (25 μM) in the presence of TTX (1 μM). ^*^p < 0.05, ^**^p < 0.01, and ^***^p < 0.001. Two-tailed paired *t*-tests were used, except in panels **E, F** (Phenserine) where repeated measures ANOVA followed by Bonferroni-corrected pairwise comparison was used.

Although we have demonstrated that ACh application has important effects on GABAergic inputs to RB cells, it remains to be shown whether endogenous ACh is able to modulate A17-RB cell signaling. For this purpose we analyzed the effects of acetylcholinesterase (AChE) inhibitors on A17 and RB cell activity. Perfusion with neostigmine or phenserine induced a marked increase in the electrical charge moved during ACh-induced responses in A17 (neostigmine 2 μM, 641 ± 114% of control, *n* = 7, *p* = 0.005; phenserine 5 μM, 262 ± 22%, *n* = 4, *p* = 0.039, Figures 6A,C) and RB cells (neostigmine 2 μM, 698.8 ± 197%, *n* = 10, *p* = 0.0005, Figures 6B,C), demonstrating that AChE actively cleaves ACh in rat retinal slices. Interestingly, application of neostigmine alone was able to significantly depolarize A17 cells (neostigmine 2 μM, from −61.4 ± 0.9 to −53.2 ± 1.8 mV, *n* = 5, *p* = 0.0041). This depolarization was mediated by nAChR activation as it was reversed to near-control values by subsequent perfusion with TMPH (neostigmine 2 μM + TMPH 10 μM, −60.4 ± 1.2 mV, *n* = 3, *p* = 0.01, Figures 6D,E). Similarly, during voltage-clamp recordings from a different set of cells, AChE inhibitors induced long-lasting inward currents (neostigmine 2 μM, from 12.5 ± 11.8 pA to −20.5 ± 11.9 pA, *n* = 7, *p* = 0.012; phenserine 5 μM, 0.8 ± 7.1 pA to −24.9 ± 5.9 pA, *n* = 5, *p* = 0.04, Figure 6F). The observed effects of AChE inhibitors were not accompanied by an apparent increase in EPSC frequency, suggesting that nAChRs in A17 cells might be non-synaptically activated. Indeed, the frequency of spontaneous EPSCs during inhibition of AMPA/Kainate receptors (NBQX 5 μM) did not change after perfusion of a specific nicotinic antagonist (0.54 ± 0.18 Hz during NBQX vs. 0.6 ± 0.3 Hz after TMPH + NBQX, *n* = 4, *p* = 0.63, Figure S1G), showing the absence of spontaneous nAChR-mediated synaptic events in A17 cells. In RB cells neostigmine increased the frequency of spontaneous IPSCs about 5 fold (from 0.8 ± 0.1 to 3.5 ± 0.2 Hz, *n* = 4, *p* = 0.0008, Figures 6G,H). A similar result could be observed when low concentrations of nicotine were applied in the presence of TTX (Nicotine 25 μM, from 1.2 ± 0.4 to 6.4 ± 2 Hz, *n* = 5, *p* = 0.047, Figure 6I) indicating that these enhancements in IPSC frequency by nicotine and neostigmine are caused by increased GABA release from A17 cells. In summary, these results demonstrate that in the slice preparation under scotopic conditions, ACh is intrinsically released reaching concentrations which are high enough to depolarize A17 cells and boost synaptic GABAergic neurotransmission.

**Figure 7:**
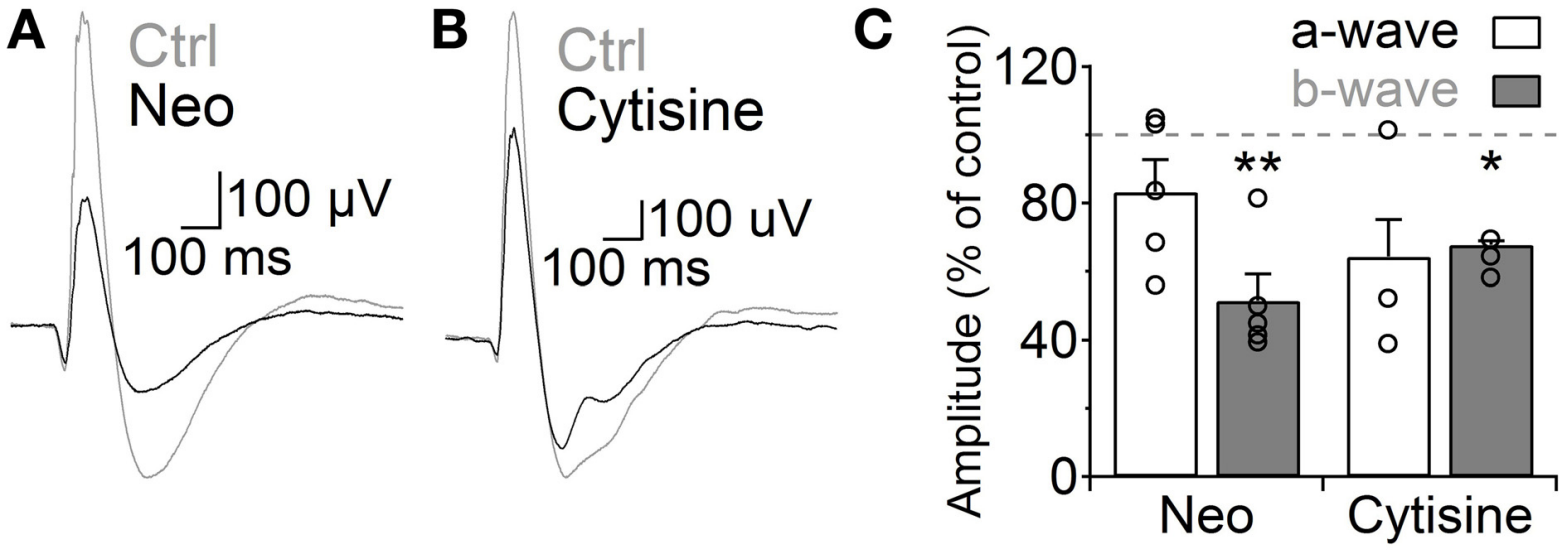
**Activation of nicotinic receptors reduces the rod bipolar-driven b-wave of the scotopic electroretinogram**. **(A**,**B)** representative traces of the scotopic flash ERG response in control conditions and after intravitreal injection of **(A)** neostigmine (25 μM) or **(B)** the specific nAChR agonist cytisine (500 μM). **(C)** Bar graph showing average changes in the scotopic ERG a- and b-waves after neostigmine or cytisine injections. Two-tailed paired *t*-test, ^*^p < 0.05 and ^**^p < 0.01.

Finally, we analyzed how the increase in GABAergic signaling mediated by ACh and nAChRs affects light responses of RB cells. We addressed this question using the *in vivo* scotopic flash-evoked ERG. Intravitreal injection of neostigmine produced a marked decrease in the ERG b-wave amplitude (Neo 25 μM, 51.5 ± 7.7% of control, *n* = 5, *p* = 0.005), which mainly represents RB cell depolarization (Weymouth and Vingrys, 2008), without significantly altering the photoreceptor-mediated a-wave (83.3 ± 9.5% of control, *n* = 5; Figures 7A,C). Similarly, injection of cytisine produced a significant reduction in the b-wave (cytisine 500 μM, 64.1 ± 1.8, *n* = 3, *p* = 0.006; Figures 7B,C) and a non-significant decrease in the a-wave (*p* = 0.199). This indicates that ACh modulates RB cell light responses *in vivo* through the activation of nAChRs.

## Discussion

Neuronal activity in the inner retina is shaped by a wide variety of GABAergic and glycinergic ACs, which form numerous inhibitory synapses with bipolar and GCs, and provide feedback and feed-forward inhibition to the inner retinal circuitry (Macneil and Masland, 1998; Wässle, 2004; Masland, 2012). The overall excitability of the network is also under the influence of neuromodulators acting on different types of cells in a slower time scale, enhancing the flexibility of the system (Marc, 2004; Witkovsky, 2004; Huang et al., 2013). RB cell output is controlled by a diverse and heterogeneous set of inhibitory inputs (Eggers and Lukasiewicz, 2006; Eggers et al., 2007; Chávez and Diamond, 2008; Chávez et al., 2010), but reciprocal synapses with A17 cells (Hartveit, 1999; Singer and Diamond, 2003; Chávez et al., 2006) are the most numerous (Strettoi et al., 1990; Kim et al., 1998). The present study describes a novel modulatory mechanism of A17 cell activity mediated by ACh and heteromeric nAChRs.

### Subunit composition of nAChRs in A17 cells

Nicotinic receptors comprise a heterogeneous family formed by different combinations of α and βsubunits (Dani and Bertrand, 2007; Millar and Gotti, 2009). Biochemical evidence suggests that retinal nAChRs are assembled as complex combinations of α_2_–α_6_ and β_2_–β_4_ subunits (Moretti et al., 2004; Marritt et al., 2005). Using pharmacological agents which are subunit-specific at the concentrations used (Alkondon and Albuquerque, 1993; Harvey and Luetje, 1996; Papke et al., 2000), we demonstrate here that A17 cells do not express the commonly found α_7_ or α_4_β_2_ nAChRs. Instead, the strong response to the agonist cytisine (Alkondon and Albuquerque, 1993; Papke and Heinemann, 1993; Takeda et al., 2003; Smith and Uteshev, 2008) as well as the complete block exerted by TMPH (Decker and Anderson, 1995; Damaj et al., 2005; Papke et al., 2005) suggest that these nAChRs contain the β_4_ subunit. Particularly, the low efficacy of DHβE in blocking ACh-induced currents and the failure of RJR-2403 to evoke responses in A17 or RB cells, indicate that nAChRs in A17 amacrine cells might be of the α_3_β_4_ subtype (Harvey and Luetje, 1996; Papke et al., 2000). This pharmacological characterization is further supported by immunohistochemical data that failed to demonstrate α_3_β_2_ or α_7_ nAChRs in rabbit A17-homologous cells (Keyser et al., 2000; Dmitrieva et al., 2007, 2003). Finally, nAChRs mediating ACh-evoked responses in A17 cells had low Ca^2+^ permeability and showed slow kinetics and weak desensitization (Figures 2, 5), in agreement with the properties of β_4_ subunit-containing nAChR channels (Papke and Heinemann, 1994; Quick et al., 1999; Takeda et al., 2003; Fucile, 2004). Therefore, our data are consistent with the interpretation that A17 cells of the rat retina express heteromeric nAChRs that contain the β_4_ subunit, although further experiments are necessary to determine the exact composition of these receptors. Interestingly, the pharmacology of Ach-induced GABA release into RB cells closely resembles that of inward currents observe in A17 AC, supporting their prevalence in this modulatory mechanism. Nevertheless, GABA release was more affected by nicotinic antagonists than A17 cell ACh-induced inward currents (Figure 3) and less sensitive to agonists (Figure 4), suggesting that a minimum depolarization of A17 terminals by ACh is required to trigger synaptic release.

### Mechanism of ACh-induced inhibition of RB cells

Reciprocal feedback to RB cells depends on calcium flow through Ca^2+^-permeable AMPA receptors (CP-AMPARs) and postsynaptic GABA_A_ receptors (Singer and Diamond, 2003; Chávez et al., 2006, 2010). BK channels present in A17 cell varicosities curtail CP-AMPAR-mediated depolarization and limit L-type VGCCs involvement in GABA release (Grimes et al., 2009). Nevertheless, strong or long-lasting glutamate drive depolarizes A17 cells sufficiently to overcome BK channel inhibition and engage VGCCs in release events (Grimes et al., 2009) that activate both GABA receptor subtypes (Hartveit, 1999; Dong and Hare, 2002a,b; Singer and Diamond, 2003; Eggers et al., 2007; Chávez et al., 2010). NAChRs can influence neurotransmitter release through a variety of mechanisms, including direct Ca^2+^ influx through the channel, recruitment of VGCCs by membrane depolarization or through intracellular Ca^2+^ release (Vizi and Lendvai, 1999; Dajas-Bailador and Wonnacott, 2004). We have shown that ACh-induced GABA release in A17 cells is mainly mediated by L-type VGCCs and CICR (Figure 5) and preferentially activates GABA_C_ receptors on RB cells (Figure 1). Cholinergic recruitment of VGCCs in A17 cells will be facilitated by the low Ca^2+^ permeability (avoiding early activation of BK channels) and slow kinetics of A17 nAChRs. Interestingly, these previous observations suggest a correlation between VGCCs-mediated Ca^2+^ entry in A17 cells and postsynaptic GABA_C_receptors. Considering that GABA_A_and GABA_C_ receptors are spatially segregated in the RB cell axon (Fletcher et al., 1998; Koulen et al., 1998; Chávez et al., 2010), it is tempting to hypothesize that there are different classes of release sites within A17 cells and that ACh induces GABA release preferentially from those presynaptic to GABA_C_ receptors. Our measurements showed that Ca^2+^ signals evoked by ACh were stronger in regions containing varicosities, following the distribution of L-type VGCCs in A17 cells (Grimes et al., 2009). It would be interesting to analyze the subcellular localization of nAChRs in A17 cells in relation to VGCCs and postsynaptic GABA receptors to better understand the mechanisms of action of this nicotinic modulation of retinal GABAergic signaling.

### Cholinergic modulation of RB cell GABAergic inhibition

In the mammalian retina, under photopic and scotopic conditions, ACh is released both tonically and in a light-induced manner (Masland and Livingstone, 1976; Massey and Neal, 1978, 1979a,b; Masland et al., 1984; O'Malley and Masland, 1993). Although currently the *in vivo* dynamics of ACh activation of A17 cells are unknown, our results support a system especially suited for sustained control of GABA release. Namely, nAChRs with sluggish kinetics gate slowly inactivating L-type VGCCs, inducing GABA release that binds preferentially high-affinity and slow GABA_C_ receptors. Indeed, our data shows that the duration of nAChR-induced depolarization of A17 cells and GABA release onto RB cells was mainly controlled by ACh availability and AChE activity (Figure 6), demonstrating that ACh can control RB cell activity on a slow time scale. Moreover, the lack of nAChR-mediated synaptic events suggests that activation of A17 nicotinic receptors occurs extrasynaptically, a common mechanism for cholinergic neurotransmission in the CNS that enables long-lasting modulation of synaptic activity (Dani and Bertrand, 2007; Lawrence, 2008; Lendvai and Vizi, 2008; Arroyo et al., 2014). In the retina, fast synaptic nAChR activation could be expected for cells directly connected to SAC output (Brown and Masland, 1999; Yamada et al., 2003; Dong et al., 2004; Fried et al., 2005), but ACh also influences GCs that do not make synaptic contacts with SACs (Ames and Pollen, 1969; Masland and Ames, 1976; Ariel and Daw, 1982; Strang et al., 2003, 2005). This suggests that volume release of ACh is an important mode of cholinergic transmission in the retina, as it is for the dopaminergic (Puopolo et al., 2001), melatoninergic (Huang et al., 2013) and nitrergic systems (Vielma et al., 2012). On the other hand, injection of AChE inhibitors into the rat vitreous reduced the b-wave of the scotopic flash ERG response in the rat, probably due to an enhanced activation of nAChRs, as it could be partially replicated by injection of a specific agonist (Figure 7). This result is in agreement with the observed effects of nicotine in humans (Jurklies et al., 1996; Varghese et al., 2011) and shows that activity of bipolar cells is actively modulated by nAChRs *in vivo*, although the exact nature and dynamic properties of this modulation require further assessment.

## Conclusion

We are still far from understanding the complexities of the retinal cholinergic neurotransmitter system as the functional relevance of the widespread expression of cholinergic receptors has been elusive, with the notable exceptions of ACh effects on direction-selective GCs (Grzywacz et al., 1998; Fried et al., 2005; Reed et al., 2005) and during development (Feller, 2002). This study demonstrates that in the adult rat retina, ACh is a major player in the regulation of GABAergic inhibition of RB cells. We hypothesize that non-synaptic nAChR activation slowly depolarizes A17 cells, which facilitates GABA release via L-type VGCCs enhancing its gain control function of the RB-AII cell synapse (Dong and Hare, 2002a,b). This cholinergic control provides A17 cells with a modulatory system independent from the activity of RB cells, their main excitatory input and exclusive output, an advantageous situation that would greatly improve the adaptability and computational capabilities of A17 amacrine cells.

## Author contributions

Claudio Elgueta designed, performed and analyzed the experiments, designed acquisition and analytical tools and wrote the paper. Oliver Schmachtenberg and Adrian G. Palacios designed the experiments and wrote the paper. Alex H. Vielma performed experiments and analyzed the data.

### Conflict of interest statement

The authors declare that the research was conducted in the absence of any commercial or financial relationships that could be construed as a potential conflict of interest.

## Abbreviations

AC: amacrine cell
ACh: acetylcholine
CP-AMPAR: calcium permeable AMPA receptor
CICR: calcium induced calcium release
GC: ganglion cell
IPL: inner plexiform layer
nAChR: nicotinic acetylcholine receptor
RB: rod bipolar.
