# Supplementary material for: Acetylcholine induces GABA release onto rod bipolar cells through heteromeric nicotinic receptors expressed in A17 amacrine cells
Source: Front Cell Neurosci. 2015 Feb 9;9:6. doi: 10.3389/fncel.2015.00006 (PMC4321611; doi:10.3389/fncel.2015.00006)
Supplement: Supplementary file 1 [file Table1.DOCX]

| **Rod bipolar cell** | **Control (pA)** | **Test (pA)** | **n** | **%** | **p** | **% of recovery (n)** |
| --- | --- | --- | --- | --- | --- | --- |
| SR 10 μM | 31.8±4 | 25±4.7 | 12 | 77±6 | 0.008 | 109.8±15.6(8) |
| TPMPA 50 μM | 23.2±4.7 | 5.6±0.6 | 10 | 29±3.3 | 0.0025 | 65.7±8(4) |
| TPMPA+ SR | 27±6.3 | 1.5±0.3 | 8 | 6.2±1.4 | 0.0052 | 99±14(3) |
| NBQX 5 μM | 27.2±6.6 | 25.4±5.8 | 5 | 94.2±2.9 | 0.2 | 100.3±0.8(2) |
| TTX 1 μM | 13.8±3.4 | 12.5±1.6 | 6 | 93.7±12.2 | 0.21 |  |
| Mecamylamine 2 μM | 19.6±5.4 | 0.9±0.2 | 4 | 6.7±3.6 | 0.039 | 65.4±7.5(3) |
| Scopolamine 10 μM | 20.6±5 | 20.4±6 | 6 | 94.8±9.9 | 0.91 |  |
| TMPH 10 μM | 20±3.9 | 0.6±0.4 | 5 | 3.2±0.4 | 0.0079 |  |
| MLA 10 nM | 17.5±4.4 | 16.8±4.1 | 8 | 96.7±2.7 | 0.17 |  |
| DHβE 10 μM | 24.4±3 | 15.1±2.5 | 6 | 61±5.3 | 0.0029 | 82±5.2(6) |
| Erysodine 10 μM | 23.6±3 | 3.7±0.6 | 10 | 17±0.8 | 0.00003 | 54±1.3(7) |
| Choline 1 mM | 7.5±2 | 0.2±0.3 | 5 | 2.5±2 | 0.017 |  |
| RJR 100 μM | 25.3±7.7 | 0.35±0.05 | 3 | 1.7±0.6 | 0.004 |  |
| Cytisine 100 μM | 29.2±7.4 | 29.5±8 | 5 | 113.3±22.1 | 0.97 |  |
| 0 Ca^2+^ | 28.2±7.1 | 9.5±3.6 | 4 | 29.5±6 | 0.015 | 94±10.2(3) |
| 0 Ca^2+^ + EGTA 1 mM | 27.3±8 | 7.4±3.2 | 4 | 25.3±5.2 | 0.003 | 100.3±27.2(3) |
| CdCl_2_ 200 μM | 17.7±3.3 | 2.2±0.5 | 9 | 14.5±2.8 | 0.001 |  |
| CoCl_2_ 1 mM | 14.7±2.8 | 3.1±0.9 | 4 | 19.5±1.2 | 0.01 | 71.8(1) |
| Verapamil 20 μM | 18.8±6 | 1.1±0.2 | 5 | 7.3±1.4 | 0.019 | 82.1±14.1(2) |
| Nifedipine 30 μM | 9.7±9 | 1.6±0.3 | 5 | 17±3.7 | 0.0007 | 95.3±5.5(2) |
| Ruthenium Red 40 μM | 35.6±1.7 | 10±2.7 | 5 | 26.7±3 | 0.0024 | 75±10.6(5) |
| CPA 30 μM | 7.1±2.1 | 2.5±0.8 | 4 | 35.5±5.6 | 0.043 | 68±31.5(2) |
| 4-CMC 500 μM | 17.1±4.7 | 8.2±2.1 | 4 | 50.4±6.3 | 0.049 | 83.2±7.1(3) |
|  | | | | | | |
| **A17 amacrine cell** | **Control(pA)** | **Test (pA)** | **n** | **%** | **p** | **% of recovery (n)** |
| CdCl_2_ 200 μM | 323.8±81.3 | 323.9±76.6 | 5 | 100.9±5.7 | 1 |  |
| CoCl_2_ 1 mM | 148.5±60 | 150.8±63.7 | 4 | 98.6±4.1 | 0.65 |  |
| NBQX 5 μM | 463.8±31.1 | 542.4±40 | 3 | 116.5±8.6 | 0.42 |  |
| Mecamylamine 2 μM | 222.2±28.4 | 35.5±7 | 17 | 15.4±1.5 | 0.00004 | 75.9±7.7(12) |
| Scopolamine 10 μM | 111.4±26 | 119.2±28 | 4 | 107.4±12.4 | 0.58 |  |
| TMPH 10 μM | 385±93.4 | 11.9±2.8 | 5 | 3.6±0.7 | 0.019 | 28.5(1) |
| MLA 10 nM | 176.1±26.7 | 171.1±26.2 | 8 | 96.8±4.2 | 0.25 |  |
| DHβE 10 μM | 214.3±23 | 190.3±21.5 | 15 | 89.7±3.1 | 0.0086 | 96.8±3(7) |
| Erysodine 10 μM | 246.3±36.9 | 84.7±15.9 | 10 | 36.1±3.3 | 0.0002 | 93.1±7.4(5) |
| Choline 1 mM | 271.9±80.3 | 26.9±9.9 | 5 | 10.1±3.9 | 0.03 |  |
| RJR 100 μM | 354.5±49.3 | 23.8±14.4 | 4 | 7.5±5 | 0.009 |  |
| Cytisine 100 μM | 168±53.5 | 180.5±61.4 | 5 | 108.7±14.7 | 0.58 |  |

**Table 1.** Summary of response amplitude evoked by acetylcholine or nicotinic agonists, in control conditions, during the application of different drugs that modify pre or pos-synaptic response (Test column), and after washout of the tested manipulation. Upper and lower sections show responses obtained in rod bipolar cells and A17 cells respectively. Values are presented as mean ± standard error. % represents the average percentage of the response after the treatment and n represents the number of experiments done for the tested condition. The p-value was obtained doing two-tailed paired t-tests from the respective control situation.
